# Paediatric Psychosocial Emergencies in Two Inner-City London Hospitals: Review of the Current Management and Critical Evaluation Using NICE Self-Harm Quality Standards (QS34)

**DOI:** 10.1192/bjo.2022.324

**Published:** 2022-06-20

**Authors:** Mikail Ozer, Isil Thrasher, Raj Sekaran

**Affiliations:** 1 Barnet Enfield and Haringey Mental NHS Health Trust, London, United Kingdom; 2 Camden and Islington NHS Foundation Trust, London, United Kingdom

## Abstract

**Aims:**

The paediatric wards support many children presenting with psychosocial crises. This has been increasing in recent years. NICE quality standards recommend that children who have self-harmed receive: a comprehensive psychosocial assessment, are assessed within 24 hours of referral if at high risk of suicide, a collaboratively developed risk management plan and monitoring to reduce risk of further self-harm. We aim to measure the number of referrals made by hospitals for acute psychiatric presentations and the adherence to the above quality standards by the Service for Adolescent and Families in Enfield.

**Methods:**

We retrospectively audited inpatients referred by North Middlesex hospital and Barnet hospital. Referral data were collected over 5 years. Data collected between April 2018 and March 2019 were evaluated to review good practice and adherence to the NICE quality standards. For each patient, we collected data on whether they have had a comprehensive psychosocial assessment, if the assessment was completed within 24 hours, 7-day follow-up review and a documented risk assessment.

**Results:**

There has been a 141% increase in hospital referrals to the service from 2014/15 to 2018/19. The service had 130 referrals between April 2018 and March 2019. 72% of referrals came from North Middlesex hospital and 28% were from Barnet hospital. Ages were between 5 and 18. Girls formed 74% of all presentations. 87% of patients presented with deliberate self-harm, suicidal ideation or suicide attempt. Of all referrals 100% had a comprehensive psychosocial assessment, 93% were seen within 24 hours of being referred, 97% had a documented risk assessment and 92% had a 7-day follow-up review.

**Conclusion:**

Self-harm and suicidal ideation in children are rising, especially among girls aged 13 to 16 years (increased by 68% between 2011 and 2014). The gender inequality in our referrals further supports these findings. Higher rates of self-harm have been shown in more deprived areas and could be associated with gang involvement, bullying, abuse, gender identity and family issues. We have developed an assessment protocol and safety plan, are liaising with hospitals daily to arrange assessments and follow-up. Paediatric nurses have been trained in the time to talk programme and a full-time crisis liaison nurse has been employed. This will be re-audited to measure effectiveness of interventions.

